# Exploring meaning in culture: Insights from meaning-centered psychotherapy in Aotearoa, New Zealand

**DOI:** 10.1017/S1478951526102788

**Published:** 2026-06-03

**Authors:** Cerys Clayden, Alesha Wells, Allison J. Applebaum, Lisa Reynolds

**Affiliations:** 1 Department of Psychological Medicine, Faculty of Medical and Health Sciences, University of Auckland, Grafton, Auckland, New Zealand; 2 Māori iwi affiliation to Ngāti Awa; 3 Brookdale Department of Geriatrics and Palliative Medicine, Icahn School of Medicine at Mount Sinai, New York, NY, USA

**Keywords:** MCP, Indigenous, culture, meaning, cancer, psychedelic-assisted therapy

## Abstract

Meaning-Centered Psychotherapy (MCP) has shown significant promise in reducing existential distress and improving wellbeing in people with advanced cancer. Research is yet to explore the applicability and translation of MCP into an Indigenous Aotearoa New Zealand cultural context.

**Objectives:**

This research sought to explore the applicability of MCP within Aotearoa New Zealand. The objectives of this study were to investigate Indigenous patients’ experiences of an MCP trial and Indigenous therapists’ experiences of recruitment and MCP delivery within the trial.

**Methods:**

Semi-structured interviews were conducted with 2 Indigenous patients with advanced cancer receiving MCP, 1 Indigenous support person, and 4 Indigenous health psychologists delivering MCP about their experience in the research trial. Participants were also asked their views on recruiting Indigenous populations into research trials, and on the applicability of MCP in Aotearoa New Zealand. Reflective thematic analysis was utilized to analyze interview transcripts.

**Results:**

This study showed that trust is central to recruiting Indigenous populations into research trials and contributed to the overall success of MCP delivery. Participants highlighted community trust and organizational mistrust as potential contributors toward recruitment challenges. Trust in the self, in others, and in culture was central to finding meaning through MCP. When conducted in a safe patient-centered therapeutic space, MCP concepts and meaning-making can integrate effectively into Te Ao Māori and an Indigenous context.

**Significance of results:**

MCP concepts and delivery may integrate well into a Te Ao Māori framework. Care should be taken in future delivery of MCP within Aotearoa New Zealand, ensuring the patient-focused nature of the therapeutic modality is maintained, and the patient’s own connection with culture is the highest priority.

## Introduction

Advanced cancer is often associated with existential distress (Arrieta et al. 2013; Philipp et al. 2025), described as feelings of hopelessness and a loss of meaning and purpose in life (Masterson et al. 2018; Applebaum 2019). Such distress can increase suicidal ideation and desire for a hastened death (McClain et al. 2003; Zhang et al. 2024). Pharmacotherapeutic and psychotherapeutic approaches aimed at reducing existential distress have shown varied efficacy and uptake in cancer populations (Grassi et al. 2014; Brebach et al. 2016). Current limitations in treatment options drive a need for effective and accessible therapeutic approaches, with appropriate consideration of Indigenous contexts.

As a colonized nation, the Indigenous population of Aotearoa New Zealand (henceforth Aotearoa) has faced historical policies of assimilation, cultural suppression, and loss of land (Awe-Bevan 2013). The ongoing forces of colonization have entrenched the disadvantage faced by Indigenous Māori, including inequities in social and structural determinants of health, leading to disparate health statistics. Māori are more likely than non-Māori to receive a cancer diagnosis (Ministry of Health 2018; Gurney et al. 2025) and are less likely to survive the disease (Teng et al. 2016). This is due to several factors, including barriers to accessing early detection screening and receiving high-quality care, exacerbated by institutional racism (Gurney et al. 2025). Despite experiencing greater distress and reduced quality of life, uptake by Māori of supportive cancer care services in Aotearoa is also lower (Xiao et al. 2025). Evidently, Western healthcare models fall short in adequately meeting the needs of Māori. Therefore, it is crucial to improve access to culturally tailored supportive care interventions to reduce, rather than exacerbate, health disparities in this population.

Meaning-Centered Psychotherapy (MCP) has shown significant promise in reducing existential distress and improving wellbeing in people following a cancer diagnosis (Breitbart et al. 2015, 2018). The experience of meaning in life can arise from values and beliefs that provide purpose (Puchalski and Romer 2005; Shoji et al. 2018), and an enhanced sense of meaning and purpose can buffer against existential distress and suicide risk (Zhang et al. 2024). Influenced by the work of Viktor Frankl (1985) and originally formulated as a group therapy for patients with advanced cancer in the United States (Breitbart et al. 2018), MCP has since been adapted to be delivered individually in 7 sessions and aims to help patients connect – or reconnect – to various sources of meaning in life that can serve as resources to help one cope with challenges, limitations, and losses (Breitbart et al. 2018). Meaning-centered interventions have been shown to be helpful in cancer patient populations, improving quality of life and social relationships (Teo et al. 2019), and reducing depressive symptomology (Shen et al. 2025).

Psychotherapeutic interventions targeted to specific cultural communities yield more efficacious outcomes than treatments not culturally adapted (Griner and Smith 2006; Smith et al. 2011). Internationally, there is a growing body of research investigating the applicability of MCP for diverse cultures and ethnicities (Leng et al. 2019; Costas-Muñiz et al. 2020). In the United States, researchers have adapted MCP to serve Spanish-speaking Latino patients (Costas-Muñiz et al. 2016; Blasco et al. 2022) and Chinese immigrants (Leng et al. 2016). Within these adaptations, cultural considerations were regarded, including language, context, content, therapeutic relationships, and goals (Costas-Muñiz et al. 2016, Leng et al. 2018, 2019). Spirituality is a fundamental backbone for wellbeing within many non-Western health models, with adaptations of MCP for Latino and Chinese populations prioritizing spirituality as a vehicle for healing, strength, and meaning (Costas-Muñiz et al. 2016; Leng et al. 2016). Cultural and linguistic adaptations of MCP have been shown to be acceptable to minority populations when tailored to their language and spiritual needs (Costas-Muñiz et al. 2020; Lui et al. 2025). Research with Puerto Rican advanced cancer patients found that family-oriented contexts for death and related concepts were more acceptable than standard MCP explanations of meaning and legacy (Torres-Blasco et al. 2021; Blasco et al. 2022). A correlate cultural adaptation of MCP for Māori has not yet been undertaken, and given the potential benefits of MCP for this population, it warrants future investigation.

To date, only 1 clinical trial delivering MCP in Aotearoa has been conducted (Psychedelic-Assisted Meaning-Centered Psychotherapy; PAM Trial; see Wells et al. 2024). The current study sought to qualitatively explore the experiences of patients receiving MCP in this trial, and of therapists delivering MCP within this trial to guide future MCP practice in Aotearoa. This study also aimed to shed light on the recruitment challenges faced by the parent trial, which did not achieve equal representation of Māori and non-Māori participants, as promoted by responsivity frameworks (Reid et al. 2017).

## Methods

As outlined above, this study was conducted as part of a parent trial investigating the feasibility, acceptability, and safety of lysergic acid diethylamide (LSD) microdosing alongside MCP in patients with advanced cancer (Wells et al. 2024). In recognition that Māori participation in such study is critical for adequate representation (Haft et al. 2025), the PAM trial aimed to recruit a sample that comprised of 50% Māori through several responsive techniques. However, the trial was closed prematurely due to unexpected difficulties in sourcing the investigational drug, and, at that point, Indigenous representation was not met. A study extension was conducted where participants received MCP, but no drugs were administered. The purpose of this extension was to investigate Indigenous patients’ experiences of receiving MCP and Indigenous therapists’ experiences of delivering MCP in an Aotearoa context. This work also sought to investigate barriers to participation in MCP clinical trials for Māori and Indigenous communities. A qualitative approach was chosen to garner an in-depth understanding of the experiences, perceptions, and applicability of receiving and delivering MCP in the trial.

### Participants

Participants were either 1) Indigenous cancer patients participating in the no-drug PAM Extension Trial, or 2) Indigenous psychologists with experience delivering MCP on the PAM Trial.

### Procedure

Patient participants in the PAM Extension trial were recruited through social media advertisements and oncologist referrals. Psychologists who had delivered MCP in the PAM trial were recruited through direct email invitation.

Semi-structured interviews lasted approximately 1 h and were conducted in person or via videoconferencing software (Zoom). Patient participants of the PAM Extension Trial were interviewed 1 month following their last MCP session. Psychologists were interviewed after their involvement in the trial had been completed. Interviews were conducted from 21 October to 14 November 2025. All interviews were recorded using secure recording software.

Interviews began with an introduction and an opportunity to ask questions. Participants were given the option to open and close the interview in a meaningful way for them, with the interviewer offering karakia (Māori prayer). A semi-structured interview guide facilitated questioning. All interviews were transcribed verbatim by CC, manually transcribing kupu Māori (Māori words) used by participants to ensure accurate transcription. In keeping with Indigenous data sovereignty (West et al. 2020), recordings and transcriptions were stored on a local secure server rather than international cloud servers. Transcripts were de-identified with assigned participant numbers.

### Analysis

Reflexive thematic analysis was employed to flexibly provide a rich interpretation of experience and perspective (Braun and Clarke 2006). A member of the research team (CC) initially familiarized themselves with transcripts, assigning codes to salient semantic (explicit meaning) and latent (underlying ideas) messages. Through an iterative process, the research team collated codes into linked themes. Themes were collaboratively refined and developed with all researchers before being defined and labelled.

## Results

Patient participants (*N* = 2) in this study were aged 50–65 years, identified as male (*n* = 1) or female (*n* = 1), were Māori (*n* = 1) or Pasifika (*n* = 1), and were diagnosed with advanced-stage lymphoblastic leukemia (*n* = 1) or prostate cancer (*n* = 1). One patient participant had a family support person present who offered emotional support and contributed to the interview. Psychologist participants all had at least 3 years’ experience delivering MCP (*N* = 4) and were female registered psychologists of Māori (*n* = 2) and Pasifika (*n* = 2) descent.

### Barriers to recruiting Indigenous participants into research trials

**Figure 1.**
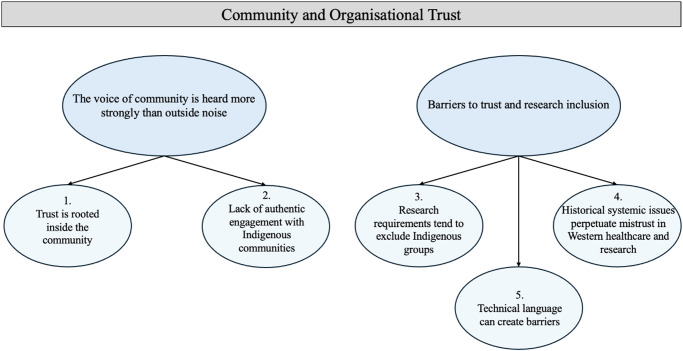
Themes related to recruiting Indigenous peoples for large research trials. Figure 1 long description.

Figure 1 presents themes related to the barriers identified in recruiting Indigenous participants to research trials. Two themes address the larger concept that the voice of the community is heard more strongly than outside voices. The other themes speak to barriers for people in trusting research. Overall, there was a narrative of trust felt strongly within communities and wariness toward research organizations.

#### The voice of the community is heard more strongly than outside noise

Participants expressed that community-based information sources are more trusted, hold more salience, and are held with higher regard than outside voices. Overall, participants noted a lack of information and research understanding within these inside circles (see Table 1).

1. *Trust is rooted inside the community* speaks to how many Indigenous peoples prefer to seek help from trusted community members before reaching for outside support. Participants spoke about how, within Indigenous circles, they have their own kaitiaki (guardians). Community and connection are strong with Māori and Pasifika, and connecting kanohi ki te kanohi (face-to-face) to establish rapport is essential. Participants reported that recruitment and recommendations for research studies need to come from trusted community sources.

**Table 1.** Representative participant quotes of subthemes developed under community and organizational trust Table 1 long description.

The voice of the community is heard more strongly than outside noise.
**Trust is rooted inside the community:**
*“I suppose they like to keep lots of these things in-house and, you know, Yeah, I suppose it’s what their beliefs are as well. They might have their own katiaki [guardian/caregiver] they refer to or… it’s a hard one.” (P1)*
*“The only thing to do is to actually go through the community providers. And that would be the best, the people who are already relating to these people. If you come through somebody that they trust, they’re probably more likely to use that service, especially if it’s the medical profession.” (P2)*
*“It needs to come from people that they trust. And I think that Pasifika and Māori are really strong knit in their communities and their family dynamics… it needs to have people in those communities understanding what’s going on, and then that trust built.” (P2S)*
*“If you were a trusted person and spoke about it, like they do about rongoā [Māori healing], yeah, then there would have been an opening… So it’s about being, talking with them a tinana [in person], kanohi ki te kanohi [face to face], which is we need to be actually present with them to actually talk about it and invite them.” (PSYC1)*
*“Kind of approaching Māori and Pacific clinicians to help recruit. But just having kind of key people in the community as well to help promote it. Like there’s a lot of kind of Māori, health-based organisations that I think could be approached.” (PSYC3)*
**Lack of authentic engagement with Indigenous communities:**
*“I think it’s a work in progress. I mean, meaning centered psychotherapy isn’t known out there. How do we promote more meaning centered psychotherapy? ...but let’s have a let’s have a conversation. Let’s have a hui [meeting] about it. All those who are interested, how are we going to get this out here?” (PSYC1)*
*“And I think more widely it would be great to have more clinicians to bounce ideas off and, peer supervised. I guess the barrier too is just accessing training to begin with to then create enough of a group of people who have expertise in it. So I guess that could be future directions is getting more people skilled in that therapeutic approach to then create a community of therapists who can then deliver.” (PSYC2)*
*“I think maybe that trust, like it being endorsed by, by healthcare professionals maybe that already have that rapport with potential participants. And to demystify it a little bit, because I do feel like there’s a very particular subset of patients who, just by looking at the name of the study, would feel interested. And then there’s a few other subsets that would just be, I don’t know what that is.” (PSYC4)*
**Barriers to trust and research inclusion.**
**Research requirements tend to exclude Indigenous groups:**
*“Māori were happy to do that. But then they didn’t fit the criteria. So we’re all happy to do it, but we can’t, if we don’t fit the criteria, we don’t fit the criteria. But that was for the research study… And when Māori don’t get anything out of it, they don’t turn up. If people don’t get what, what they feel they require or need or should have, then they walk.” (PSYC1)*
*“But it was just quite tricky with the very strict eligibility criteria. I didn’t have any patients who I was seeing in my work meet the eligibility criteria, unfortunately. So I would say it was quite challenging to start off with because the actual sample or potential sample pool is already quite low.” (PSYC3)*
*“I definitely think that the commute is a tricky one because it’s kind of like, it’s almost a full day of commitment. But it definitely means that I’ve got to block out that whole day just for these sessions. And then you never know with traffic as well and how much money is in the bank account for fuel.” (P2S)*
**Historical systemic issues perpetuate mistrust in Western healthcare and research:**
*“I guess Islanders and Māori, they tend to have a… it’s not a disrespect for the medical profession… I guess it’s more a mistrust of the medical profession from where they come from, you didn’t really have doctors, and you had witch doctors, and they weren’t the best. So people are very wary of the medical profession, and then certainly not of the understanding of the therapy.” (P2)*
*“So, there is a general mistrust, right, and for good reason, there’s been misuse of data and abuse within care that has led to these general beliefs around information, how that’s shared, how will this actually benefit our people? And I think this is likely just more a systemic issue that requires more systemic interventions.” (PSYC2)*
*“There is always the challenge of recruiting Māori and Pacific people into research in general. You know, the past, past experiences where which have maybe led to distrust in those communities because of the intent of the research, maybe the data and how it’s been used and maybe, whether there was actual reciprocity in contributing.” (PSYC3)*
**Technical language can create barriers:**
*“I think it is some big words. I remember reading it and I just, I didn’t even comprehend that meaning was in the sentence. And so it did take a little bit to digest. And I think from a cultural perspective, the big words can be really intimidating. Because psychotherapy I wouldn’t think it’s very easy for people to understand… Therapy, I think is fearful enough and then you add the psycho on top of it and like the other words, I think it’s a lot to take in.” (P2S)*
*“I think for us, because we like reading and history and these kinds of true stories, reading Viktor Frankl was quite easy for us. But I don’t think it’s accessible for Pasifika or Māori, and I don’t think it’s really relatable for them.” (P2)*
*“Some of the concepts are higher level, and even in trying to describe them as a clinician, I think the language was challenging. And then there’s also just like the challenges in how to describe spiritual experiences like transcendence, for instance. So yeah, I think the language can be challenging as a therapist to describe and then also for everyday people to understand.” (PSYC2)*
*“Some of the words like transcendence and that kind of thing, some of the words may not quite fit well with some of the communities we work with, or may not be well understood. Because obviously, transcendence even, even people who have high levels of literacy may not really understand that.” (PSYC4)*

*Note*: (P#) = Patient; (P#S) = Patient Support Person; (PSYC#) = Psychologist.
2. *Lack of authentic engagement with Indigenous communities* describes the limited information accessed by and targeted toward both Indigenous patients and psychologist communities. Psychologists reported that MCP is not as well-known as other therapeutic modalities and that increased access to training, resources, and skill development is required in Aotearoa. Some psychologists suggested hui (meetings) to discuss how to promote and increase awareness of MCP within communities and among trusted stakeholders and Indigenous healthcare providers.

#### Barriers to trust and research inclusion

Participants recognized wariness toward large organizations and research trials, acknowledging previous misuse of Indigenous knowledge. Participants recognized that although strides have been made to better represent Indigenous communities in research, inclusion is still insufficient. Low historical trust in the research process, researchers, inclusion criteria, and language proficiency contribute to the ongoing difficulty in recruiting Indigenous communities into trials.

1. *Research requirements tend to exclude Indigenous groups.* Participants noted that Māori were sometimes willing and eager to participate but often did not meet eligibility criteria due to exclusion criteria (e.g., family history of schizophrenia or other psychotic disorders, diabetes mellitus, current suicide risk). Some participants noted that Indigenous peoples may not reside within a practical distance to the treatment center, and as such, travel and timing were major barriers to participation. Psychologists urged that in future large trials, loosening of strict eligibility criteria is required to ensure accessibility for Indigenous communities.
2. *Historical systemic issues perpetuate mistrust in Western healthcare, and research* represents the ongoing impact of colonization on entrenched mistrust felt by Māori. Participants noted that Māori populations have a general mistrust of research institutions, noting past misuse of data, abuse within care, and lack of reciprocity as key contributors. One participant noted that, despite efforts to ease distrust, the underlying mistrust of the healthcare system may require more systemic intervention.
3. *Technical language can create barriers*, exposing the detrimental impact that the use of technical jargon can have on participation. Participants referred to terms such as “psychotherapy” as intimidating to those not familiar with such language. Patients noted that reading Viktor Frankl’s *Man’s Search for Meaning* (1985) would be challenging for those with lower literacy levels, and they would need support during reading. Psychologists explained that higher-level concepts such as “transcendence” were difficult to explain during sessions and that some patients had difficulty understanding them.

### The experience of delivering and receiving MCP in Aotearoa

**Figure 2.**
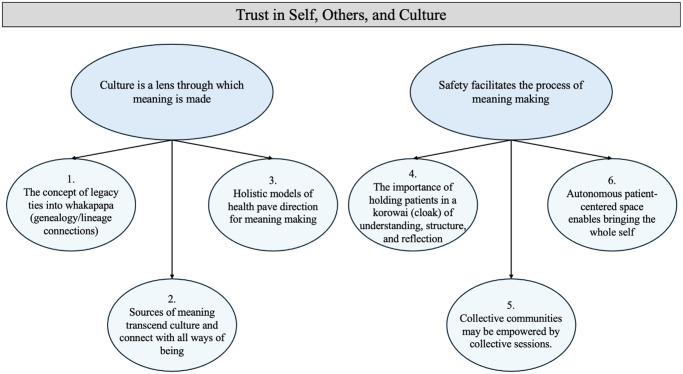
Themes regarding the experience of delivering and receiving meaning-centered psychotherapy in Aotearoa. Figure 2 long description.

Themes related to the experience of delivering and receiving MCP are represented in Fig. 2. The first 3 themes address how culture is a lens through which meaning is made. The last 3 themes acknowledge that a safe therapeutic environment facilitates the process of meaning-making. Overall, these themes represent feelings of trust in self, others, and their own culture.

#### Culture is a lens through which meaning is made

Regardless of background, participants described how culture can be a source of meaning, describing how sources of meaning for the individual mesh with holistic models of health. Participants spoke about how a person’s culture is an inherent part of them, and that when they trust their cultural connection, it can be a lens through which they pursue meaning (see Table 2 for participant quotes).

1. *The concept of legacy ties into whakapapa (genealogy/lineage)* describes how participants saw parallels between MCP and te ao Māori (the Māori worldview). Patients described exploring legacy as one of the most influential aspects of MCP. Psychologists spoke about how the concept of legacy follows whakapapa, allowing a person to bring themselves forward, supported by their culture and placement in the world. Participants reported that connection with the past can be accessed through writing a legacy project (one of the MCP exercises) and can aid people in connecting with their whakapapa.

**Table 2.** Representative participant quotes of subthemes developed under trust in self, others, and culture Table 2 long description.

Culture is a lens through which meaning is made.
**The concept of legacy ties into whakapapa (genealogy/lineage connections):**
*“I became more aware of myself. And I especially like the legacy part. I think it’s really a great part to have in it because I didn’t sort of realise how important my story was. And I think also, I think we keep referring to the good things in life, the beautiful things in life.” (P1)*
*“And I think legacy about the things that you’ve done and the people that you love and things like that. If you think along those lines, then you won’t fall back into feeling sorry for yourself and that sort of thing. So yes, each step I think gave you tools in order for you to be able to deal with it. It’s meaning in your life.” (P2)*
*“I love meaning centred psychotherapy because it’s about that whole person. It is about the acknowledgment of who they, that who they are is a culmination of their generations, you know, their ancestors, and that’s that legacy given. All that legacy present and all that.” (PSYC1)*
*“So, from a Te Ao Māori point of view, they can be their whole selves, supported by their culture to experience it in whatever way they want to take it. As well with Whakapapa [genealogy/lineage connections] being a prominent way in which Māori understand their placement within the world. Yeah, legacy is a way that can highlight that for people. Whakapapa [genealogy/lineage connections] versus, well, still legacy, but sort of use it interchangeably so that it can take more root.” (PSYC2)*
**Sources of meaning transcend culture and connect with ways of being:**
*“I was telling her about a story when I was out walking and I found a little green gecko. And she said, it’s a tohu. It’s a sign. And so I think everything we discussed was centred around Te Ao Māori. You know, talk about kaitiaki [guardians] and I think it made it more real, more, like you’ve seen the Gekko, and hearing that it’s a tohu [sign]. And because other psychologists sort of said, yeah, how lovely, but she took it to a different level, which I think is what Te Ao Māori is, just a different level.” (P1)*
*“And culturally, we’re basically all very similar, and especially when it comes to the meaning of life, and when you’re facing something like this [cancer], it strips away everything culturally. But I guess like the Polynesians and the Māoris have very strong cultural ways to deal with that.” (P2)*
*“We know in our culture that there’s a lot of roles we play. You know, there’s a lot of pōtae [hats] that we wear to just support others, others that are whānau [family], others who are not whānau [family]. Yeah, being there for people. But yeah, there’s a cultural understanding that brings meaning.” (PSYC1)*
*“[MCP] speaks directly to how we can gain meaning through our creative and experiential aspects to life that then often will open the conversation around spirituality, which I think a lot of other therapeutic modalities aren’t equipped necessarily to hold. But I mean… with every source of meaning, the creative sources, for instance, and how we express ourselves through our art forms, through sports, through movement, hula, kapa-haka, surfing, experiential sources of the water connecting to moana [ocean/lake]. I mean, it sort of transcends cultures, I feel. It can speak very directly to tapping into different cultures and different ways of being.” (PSYC2)*
**Holistic models of health pave direction for meaning-making:**
*“I think [MCP] covers Te Whare Tapa Whā, it covers the walls and the roof and the, and I think it also covers, like ako [learning], not only am I learning, the other person’s learning. Yeah. And it has lots of aroha [love] in it.” (P1)*
*“I think if it was to be something that they could, or kind of like a little bit more integrated into New Zealand, I think using stories, because there’s so many amazing Pasifika stories that do share that idea of overcoming hardship and finding a sense of meaning. Maybe a bit more of like a mythological story, but still I think it has those things that they can relate to a lot easier.” (P2S)*
*“This will likely require more extensive consultation and exploration, is we often refer back to, say, Viktor Frankl and some of the beautiful and wise insights that he had through the Holocaust, but also in therapy. And I wonder, are there scholars, thought leaders within Māori and Pasifika communities that we could refer to make it more relevant and meaningful from a cultural point of view of bringing in rather than seeking externally for these insights, more internally.” (PSYC2)*
*“From a cultural perspective, I feel like I could incorporate parts of my culture into what I share around what is meaningful for me… And then just using other, you know, Te Whare Tapa Whā as well. I would really have liked to have had the opportunity to be able to incorporate some of those models of health and also, I guess, explore the concept of wairua with people. Because I do feel like MCP and Māori well-being models kind of overlap quite well.” (PSYC3)*
**Safety facilitates the process of meaning-making.**
**The importance of holding patients in a korowai of understanding, structure, and reflection:**
*“It was not a meeting that I felt that I had to rush through and be finished it… I think it’s, I think it’s more structured, which is what I really like. I don’t know if it came with a booklet at the very beginning, but I found that really helpful as well, having a workbook to look for, to look at and to work towards.” (P1)*
*“I think the book was a really cool aspect to have to relate back to each session. And I think being faced with cancer, it can really kind of jade your perspective of life and forget about what’s really important. And I think a lot of the prompts that we were given each week helped us to remember life as a whole spectrum. Like okay, this might be the sadness and depression that might be felt in these moments, but remembering that there’s other things that can help shift, bring a little bit more lightness to the experience.” (P2)*
*“It’s a layering on the… You know how we speak of a korowai [cloak]? And the korowai [cloak] being such a precious taonga [treasure]. When we place the meaning-centred psychotherapy, that’s a korowai. When we have our culture, have an understanding or an aroha to your patient, that’s another korowai. And so, you know, there’ll be times when one will seep into it in the, the, the aroha [love] will seep into the korowai of the of the of the MCP…And then you’re holding your patient like that. You’re holding and keeping them warm. A korowai is a cloak, keeps you warm, keeps you protected.” (PSYC1)*
*“It was very challenging, but very growth developing for me to actually do the seven sessions in kind of keeping it regimented. But we know that meaning-centred psychotherapy can flow in and out of the meanings and all of that. So it was very disciplining myself… What you’re doing is just asking the questions. I mean, we get to session 4 and there’s that, what is a good death? You know, we can ask the hard questions because we’re taking them through things. And we’ve built a sense, they’ve built a sense of trust with us. They’re trusting us, they’re trusting the relationship we have. They’re enjoying finding out what they don’t usually have time or the opportunity to actually reflect on, think about, to even speak about, to even bring it to acknowledge those things that are really important to them.” (PSYC1)*
*“So it gave, or meaning-centered psychotherapy gave a wonderful structure and sense of security for that reason in being able to confidently move through existential concerns. And it was, I felt like a really strengths-based approach as well in looking at where people are and what brings them meaning versus prescribing.” (PSYC2)*
**Collective communities may be empowered by collective sessions:**
*“Everybody’s included because I know that you said that you can invite members of the family as well to talk and listen. And I think that’s another thing that, yeah, psychologists don’t do a lot. They don’t say, oh, you know, bring family, bring somebody. I think it’s a very, it can be a very family orientated hui [meeting] for, you know, for bigger meets.” (P1)*
*“I found it good to bounce off her as well. It sort of helped to bring up other subjects which I may not have otherwise brought up… But it’s certainly beneficial to have somebody else in the room with another whānau member.” (P2)*
*“I almost feel like, okay, yes, there’s the person with the cancer, but then there’s also the people alongside the people going through that journey that need this just as much… we’re all going through this journey together. And I think as well like with our communities, our wider islander communities, there is always that pillar, that person that people come to for these kinds of experiences. And I think that’s what we’re really lucky is like, we do fill those roles. But that person also needs support as well in going through that journey. Yeah, definitely making it a lot more inclusive for everyone that’s kind of walking alongside them.” (P2S)*
*“I guess encourage people to talk to their whānau [family] about what they share during the session. Potentially, if there may be scope for whānau to attend the sessions or for it to be offered to them as well if they were interested, maybe separately.” (PSYC3)*
*“And at this time we’ve got the IMCP and there’s the whānau one as well, or the family. And how do we make that accessible too? Because, you know, well we say that it’s Aotearoa in itself is actually getting more collective. We’ve got immigrants who are from collective societies.” (PSYC1)*
**Creating an autonomous patient-centered space enables bringing the whole self:**
*“It’s more accountable to not only to the psychologist, but also to the patient, because I had my part that I had to do. Yeah. And I think sometimes patients aren’t given that, that right to be part like that, have that little bit of power as well, have that sort of, or be empowered.” (P1)*
*“She was, she managed to relax you before the session, you know, and with the Karakia [prayer] and just a very gentle way of speaking… We always enjoy that sort of thing that sort of, spiritually calms things down. Even though it’s not our culture, it doesn’t matter. As far as we’re concerned, it’s the Indigenous culture of the country we’re in.” (P2)*
*“There was a few times where we would just be chatting away and then feeling like it had gone off topic, but I think it was a nice reminder the way [psychologist] would kind of bring us back to whatever it is that we are talking about, whatever’s coming up to the forefront, is meaningful for us. And that’s what this is all about.” (P2S)*
*“There’s three things we do is korero [talking], karakia [prayer], and katakata [laughter]… The korero, the katakata, the nuances of understanding. All of those things, those understandings, those are all additions. You know, that’s a cultural, an overlaying of culture.” (PSYC1)*
*“There was a lot of times I admired the patients for their openness because as you get, you know, there was, by the fact that they wanted to do this and knowing it was seven sessions and, you know, that they were courageous.” (PSYC1)*
*“I know that when I delivered it, I offered, you know, karakia [prayer] at the start and finish. So that was a lovely opportunity to do that and just an easy way to, acknowledge someone’s culture as well as my own.” (PSYC3)*

*Note*: (P#) = Patient; (P#S) = Patient Support Person; (PSYC#) = Psychologist.
2. *Sources of meaning transcend culture and connect with ways of being* paints how some patients utilize culture to connect with sources of meaning. Participants spoke of how experiential and creative sources of meaning can be found through connecting with culture, such as finding tohu (signs) in life, connecting with movement such as kapa haka (dance), and connecting with moana (ocean/waterways). Participants noted that individual sources of meaning transcend culture, with people able to experience meaning across any cultural background. Psychologists described how individuals can have many cultural roles within communities, which bring meaning.
3. *Holistic models of health pave direction for meaning-making* acknowledges how an individual can experience health through multiple sources. Participants described how MCP can be delivered to reflect holistic views of health, consistent with Indigenous perspectives. Social, spiritual, physical, and mental health can all be strengthened by focusing on an individual’s sources of meaning.

#### Safety facilitates the process of meaning-making

Participants spoke about how they needed to have trust in themselves and the person they were working with. Patients described finding MCP beneficial for their wellbeing, and for the wellbeing of their support systems. Psychologists described finding personal and interpersonal benefits from delivering MCP. Overall, participants spoke about the importance of having a safe, comfortable therapeutic space where the patient, psychologist, and support people could bring their whole selves into the therapy.

1. *The importance of holding patients in a korowai (cloak) of understanding, structure, and reflection* addresses how MCP creates a safe and trusting environment surrounding the patient. Patient participants described not feeling rushed and enjoying the routine of sessions. Psychologists reported finding it challenging to adhere to the MCP manualized structure, but gradually enjoying the “sense of security” it gave to be able to approach different topics, such as death and dying. Psychologists also described MCP as a korowai, and that through layering patients in a korowai of MCP, understanding, culture, and aroha (love) can allow a person to feel truly safe.
2. *Collective communities may be empowered by collective sessions* outlines how participants’ social wellbeing was influenced by MCP. One patient reported having a support person during sessions as beneficial, and likewise, their support person reported a meaningful experience. Psychologists reflected on social conversations that were borne from MCP outside of sessions. Participants commented on the collective nature of Māori and Pasifika communities, noting that experiencing MCP with whānau (family) is crucial so the patient is not experiencing their cancer alone.
3. *Creating an autonomous patient-centered space enables bringing the whole self* represents the therapeutic relationship MCP was able to foster. Participants reported enjoying how MCP is strengths-based and patient-centered. Patients reported feeling safe and empowered by the autonomy MCP provided to explore their own meaning. Psychologists described that MCP is patient-led and admired its flexibility in exploring meaning. Led by patient preferences, participants reported incorporating various culturally informed additions into sessions, including karakia (prayer), katakata (laughter), te reo Māori (Māori language), and casual kōrero (talk). Although not typically part of MCP, patients and psychologists described the spiritually calming nature of culturally-informed nuances in sessions.

## Discussion

This study explored the experience of recruiting, delivering, and receiving MCP in a research trial with cancer patients and therapists in Aotearoa. Our results demonstrated that trust is central to recruiting Indigenous populations and contributes to the overall success of MCP delivery. Participants highlighted organizational mistrust as contributing to recruitment challenges, urging the development of reciprocal trusting relationships between researchers and the community in future trials. Participants reported that when conducted in a safe patient-centered therapeutic space, MCP concepts and meaning-making integrate well into Indigenous contexts. These results demonstrate that MCP can be translated into practice with Indigenous peoples and environments.

### Community and organizational trust

The present study found that trust is central to recruiting Indigenous participants to clinical trials. Participants expressed that trust is more readily placed in community members, and a lack of research awareness within these communities may be limiting participation. Active engagement from community stakeholders tends to positively impact recruitment of Indigenous populations into research trials (Andrasik et al. 2021). This is consistent with a social identity framework, where individuals are more likely to exhibit trusting, altruistic behavior toward members of their in-group (Tajfel and Turner 1979; Winter and Sassenberg 2021). Review articles have noted that a lack of access to information is a recurring barrier for ethnic minority participation (George et al. 2014; Glover et al. 2015). Future research endeavors to increase Indigenous participation should actively engage with community members to demonstrate how culturally relevant knowledge and belief systems are reflected in the work. By doing so, trusting reciprocal relationships between the community and researchers are more likely to be established.

Contributing to hesitation in participation, Indigenous participants spoke about historical mistrust of research and reflected on ongoing research practices that may inadvertently cause harm (strict research inclusion criteria and academic language). Mistrust has appeared as a barrier to participation across ethnic minority research (George et al. 2014). Studies with African Americans and Indigenous Hawaiians have similarly reported mistrust related to fears of mistreatment (Scharff et al. 2010), perceptions of relinquishing rights (Herring et al. 2004), and doubts about the researcher’s agenda (Gollin et al. 2005). Within an Aotearoa context, there are historical reports of misuse of data, mistreatment, and malpractice in research (Pool 2016). High-level academic language and strict eligibility criteria act as barriers to participation, understanding, and retention for Indigenous communities (Singleton and Krause 2009; Premji et al. 2020). In aiming to improve recruitment of Māori and Indigenous communities to large research trials, future research should draw on Indigenous language, knowledge, and values to inform participation benefits and reduce mistrust in reciprocal research benefits.

### Trust in self, others, and culture

In the present study, psychologists and patients reported strong parallels between concepts from MCP and their culture. The concept of legacy resonated with Te Ao Māori (Māori worldview) concepts of whakapapa, allowing an individual to write their story supported by those who came before them. Korero whakapapa (stories told through ancestors) has a strong grounding within Te Ao Māori, with each generation assimilating their own being with the legacy of ancestral stories (Awe-Bevan 2013). Previous adaptations of MCP to Latino and Chinese immigrant populations have incorporated culture-specific concepts and metaphors into MCP sessions (Costas-Muñiz et al. 2016; Leng et al. 2018, 2019). From a local perspective, whakapapa may be used interchangeably with legacy to create a stronger affiliation with the patient. Importantly, individuals may vary in their connection to culturally specific content; one size does not fit all (Torres-Blasco et al. 2021; Blasco et al. 2022). MCP concepts of meaning allow for individual differences in cultural identity to drive meaning-making appropriate to the patient’s needs.

This study highlighted that MCP can be viewed holistically, mirroring holistic models of health. Results showed how spiritual health is integrated into MCP and can be fostered with meaningful connections to culture and faith. Research has suggested that the Te Whare Tapa Wha model (Durie 1985), a holistic Māori health model depicting 4 walls of a wharenui (meeting house) representing wellbeing, may fit well with existential psychotherapy (Wilson and Appel 2013). The literature on MCP adaptations in non-Western communities has highlighted spirituality as a primary source of strength (Costas-Muñiz et al. 2016; Leng et al. 2016). The present study mirrored the importance of spirituality for Indigenous communities and encouraged the integration of Māori concepts, stories, and kōrero whakapapa into MCP delivery. Participants highlighted the influential and impactful stories of trust, courage, and meaning through spiritual tales that may improve the connection Māori patients feel toward MCP. Research has shown the potential to interweave te ao Māori, Indigenous wisdom, and rongoā Māori (Māori healing practices) with psychotherapeutic interventions (Hanna et al. 2025; Hodge et al. 2025). MCP viewed holistically allows Māori health models to be prioritized within the therapeutic space, paving the way for meaningful connections to health through spiritual, cultural, and faith healing practices.

The present study recognized the necessity of maintaining a safe, comfortable, and supportive therapeutic environment. Our analysis showed how MCP can act as a protective korowai (cloak) of structure, reflection, and safety, enabling exploration of sources of meaning in culturally significant ways. Previous research with MCP for cancer caregivers has shown that the internal session structure and the manualized program can facilitate openness and comfort in discussing death and related concepts (Applebaum et al. 2022). Therapists are encouraged to hold MCP flexibly, following the patient’s lead while accomplishing each session’s goals (Breitbart et al. 2022). The structure of manualized MCP can facilitate conversations and comfort by providing a sense of protection to both those delivering and receiving the therapy.

Facilitating safe therapeutic environments for Indigenous communities includes supporting the wider collective nature of these communities, with the present study showing how MCP has the potential to aid not only the patient, but also their wider support systems. Previous research has shown that having support people present during psychotherapeutic interventions may improve engagement of Māori and Pasifika patients (Hirini 1997; Bennett et al. 2016). Whānau (family) involvement in therapy can buffer psychosocial distress and help patients feel equipped to continue conversations with whānau beyond treatment sessions (Bennett et al. 2014). Depending on the needs of the patient, participants of MCP should be encouraged to invite whānau members to sessions to observe, participate, and explore meaning alongside the patient.

The present study found that MCP therapists fostered a strong therapeutic alliance, allowing patients to bring their whole selves into a trusting space while maintaining autonomy over their experience. Research has shown that strong therapeutic bonds may enhance meaning-making processes during psychotherapy (Fortems et al. 2022), potentially improving therapy outcomes (Tschuschke et al. 2020). Researchers have called for the importance of intentionality in creating safe therapeutic spaces for Māori patients through whakawhanaungatanga (meaningful connection), incorporating te reo Māori (Māori language), and enhancing mana (spiritual power) in practice (Gerbic and Muriwai 2025). Other research has shown that utilizing native languages and particular language-led cultural cues within psychotherapy works as a tool to improve accessibility of care and psychological healing (Zipper-Weber 2025). Utilizing kupu Māori (Māori words) within therapeutic contexts with Māori has been shown to foster greater connection with therapeutic content when incorporated into cognitive behavioral therapy (Bennett et al. 2014) and problem-solving therapy (Hatcher et al. 2016). Maintaining a strong therapeutic alliance through patient-centered cultural session additions and autonomous patient activity may contribute to the overall success of MCP within Aotearoa.

### Implications

This study is the first to explore the experience of delivering and receiving MCP within Aotearoa for Indigenous peoples. Results showed that te ao Māori may be successfully integrated into MCP concepts and delivery. Importantly, the present study included participant voices of both Māori and Pasifika decent. Māori and Pasifika make up 17.8% and 8.9%, respectively, of the population within Aotearoa (Stats NZ Tatauranga Aotearoa 2024). While only Māori are Indigenous to Aotearoa, these groups have intersecting values, heritage, and diversity, and face similar barriers to equitable health outcomes (Matika et al. 2021). Integrating the views of both Māori and Pasifika into this study allows results to be applicable to wider communities and serve populations of Indigenous Polynesian peoples. However, the current sample size was small, and results may not generalize to other Māori, Pasifika, or Indigenous populations from other countries. Care should be taken in future delivery of MCP within Aotearoa, maintaining the patient-focused nature of the therapeutic modality by prioritizing the patient’s own connection to culture.

The present results reiterated what is widely reported in the literature, that lack of trust can be a barrier to recruitment and participation. Future research aiming to recruit Indigenous groups into clinical trials needs to focus on authentic community engagement, involvement, and applicability, including clear, culturally informed advertising and relevance. Clear and transparent data collection should always be maintained, with participants having autonomy over their information. By engaging influential community members and incorporating Indigenous concepts such as language and protocols, research can improve its relevance to Indigenous peoples and create more obvious benefits for participation.

Lack of awareness was a key barrier to participation for patients and to delivery for clinicians. Increasing the accessibility of training and peer support programmes is required to encourage the use of these concepts with patients. Clinically, MCP in Aotearoa is still in its infancy and requires broader discussion and training among clinicians, community members, and researchers to improve access to psychotherapeutic care for patients.

## Conclusion

MCP has cultural relevance to Aotearoa, with flexibility in cultural meaning-making processes and patient-centered therapy. In this study, patients and psychologists described how MCP can be understood holistically, offering space for broader models of health, cultural meaning-making, and culturally significant concepts of legacy. Participants reported that MCP can create a safe space for collective, autonomous, patient-led reflection. In disseminating MCP more widely and recruiting Indigenous populations into research trials, issues of awareness, trust, and historical mistrust will require extensive community consultation. Due to its holistic nature, MCP is well-positioned to hold the whole person as they explore meaning through a culturally significant lens.

## Data Availability

The dataset used and analyzed during the current study is available from the corresponding author on reasonable request.

## Figure 1 Long description

The diagram titled 'Community and Organisational Trust' presents themes related to recruiting Indigenous peoples for research trials. It is divided into two main sections. The first section states 'The voice of community is heard more strongly than outside noise' and includes two points: 1. Trust is rooted inside the community. 2. Lack of authentic engagement with Indigenous communities. The second section is labeled 'Barriers to trust and research inclusion' and includes three points: 3. Research requirements tend to exclude Indigenous groups. 4. Historical systemic issues perpetuate mistrust in Western healthcare and research. 5. Technical language can create barriers.

Navigate back to Figure 1.

## Figure 2 Long description

The diagram is titled 'Trust in Self, Others and Culture' and is divided into two main themes. The first theme is 'Culture is a lens through which meaning is made,' with three subpoints: 1. The concept of legacy ties into whakapapa (genealogy/lineage connections). 2. Sources of meaning transcend culture and connect with all ways of being. 3. Holistic models of health pave direction for meaning making. The second theme is 'Safety facilitates the process of meaning making,' with three subpoints: 4. The importance of holding patients in a korowai (cloak) of understanding, structure and reflection. 5. Collective communities may be empowered by collective sessions. 6. Autonomous patient-centered space enables bringing the whole self.

Navigate back to Figure 2.

## Table 1 Long description

The table compiles participant and clinician quotes about how community and organisational trust affects engagement with psychotherapy and research for Māori and Pasifika people. Quotes emphasise that trust is strongest when information and invitations come from within the community, via trusted providers, Māori and Pacific clinicians, and in-person, face-to-face conversations. Several entries describe a lack of authentic engagement, calling for hui and ongoing dialogue, more trained clinicians, and endorsement by professionals who already have rapport to reduce confusion about unfamiliar therapies. Barriers to inclusion include strict eligibility criteria that exclude willing Māori participants, limited local participant pools, and practical burdens such as travel time, traffic, and fuel costs. Historical and systemic harms are cited as drivers of mistrust, including concerns about misuse of data, lack of reciprocity, and past negative experiences with Western healthcare and research. Technical and academic language is repeatedly described as intimidating and hard to relate to, with terms like psychotherapy and transcendence seen as difficult to understand across literacy levels. Overall, the quotes suggest that culturally grounded, community-led communication and accessible language may improve trust, but the table reflects perceptions and experiences rather than measured outcomes.

Navigate back to Table 1.

## Table 2 Long description

The table compiles participant and psychologist quotes grouped into subthemes about how culture and safety shape meaning-making in meaning-centred psychotherapy. Under “culture as a lens,” patients and psychologists describe legacy as tied to whakapapa, with legacy work helping people value their story, ancestors, and life meaning. Another subtheme notes that sources of meaning can transcend culture while still being expressed through culturally grounded signs, roles, spirituality, and creative or experiential practices such as art, movement, kapa haka, and connection to water and moana. A third cultural subtheme emphasizes holistic health models, including Te Whare Tapa Whā, and suggests adapting content through Māori and Pasifika stories and local thought leaders to increase relevance. Under “safety facilitates meaning making,” quotes stress that structured sessions and workbooks create security to explore existential topics, with the korowai metaphor used to describe layered protection through therapy and cultural care. Several quotes argue that collective or whānau-inclusive sessions can support both patients and those alongside them, reflecting collective community roles. Finally, an autonomous, patient-centred space is described as empowering, using practices like karakia, kōrero, and laughter to help people bring their whole selves while keeping discussion anchored to what feels meaningful. Interpretation is qualitative and illustrative, reflecting selected perspectives rather than frequencies or outcomes.

Navigate back to Table 2.
